# Generation of patient-derived pediatric pilocytic astrocytoma in-vitro models using SV40 large T: evaluation of a modeling workflow

**DOI:** 10.1007/s11060-023-04500-6

**Published:** 2023-11-24

**Authors:** Florian Selt, Ahmed El Damaty, Martin U. Schuhmann, Romain Sigaud, Jonas Ecker, Philipp Sievers, Daniela Kocher, Christel Herold-Mende, Ina Oehme, Andreas von Deimling, Stefan M. Pfister, Felix Sahm, David T. W. Jones, Olaf Witt, Till Milde

**Affiliations:** 1 Hopp Children’s Cancer Center Heidelberg (KiTZ), Im Neuenheimer Feld 430, 69120 Heidelberg, Germany; 2 Clinical Cooperation Unit Pediatric Oncology, German Cancer Research Center (DKFZ) and German Consortium for Translational Cancer Research (DKTK), Heidelberg, Germany; 3 KiTZ Clinical Trial Unit (ZIPO), Department of Pediatric Hematology, Oncology, Immunology and Pulmonology, Heidelberg University Hospital, Heidelberg, Germany; 4 National Center for Tumor Diseases (NCT), Heidelberg, Germany; 5 Pediatric Neurosurgery Division, Department of Neurosurgery, Heidelberg University Hospital, Heidelberg, Germany; 6 Section of Pediatric Neurosurgery, Department of Neurosurgery, University Hospital Tübingen, Tübingen, Germany; 7 Department of Neuropathology, Institute of Pathology, Heidelberg University Hospital, Heidelberg, Germany; 8 Clinical Cooperation Unit Neuropathology, German Consortium for Translational Cancer Research (DKTK), German Cancer Research Center (DKFZ), Heidelberg, Germany; 9 Faculty of Biosciences, Heidelberg University, Heidelberg, Germany; 10 Department of Neurosurgery, Heidelberg University Hospital, Heidelberg, Germany; 11 Division of Pediatric Neurooncology, German Cancer Research Center (DKFZ) and German Consortium for Translational Cancer Research (DKTK), Heidelberg, Germany; 12 Division of Pediatric Glioma Research, German Cancer Research Center (DKFZ), Heidelberg, Germany

**Keywords:** Pediatric low-grade glioma cell lines, Pilocytic astrocytoma, In-vitro models, Inducible SV40 large T, Circumvention of OIS

## Abstract

**Purpose:**

Although pediatric low-grade gliomas (pLGG) are the most common pediatric brain tumors, patient-derived cell lines reflecting pLGG biology in culture are scarce. This also applies to the most common pLGG subtype pilocytic astrocytoma (PA). Conventional cell culture approaches adapted from higher-grade tumors fail in PA due to oncogene-induced senescence (OIS) driving tumor cells into arrest. Here, we describe a PA modeling workflow using the Simian Virus large T antigen (SV40-TAg) to circumvent OIS.

**Methods:**

18 pLGG tissue samples (17 (94%) histological and/or molecular diagnosis PA) were mechanically dissociated. Tumor cell positive-selection using A2B5 was perfomed in 8/18 (44%) cases. All primary cell suspensions were seeded in Neural Stem Cell Medium (NSM) and Astrocyte Basal Medium (ABM). Resulting short-term cultures were infected with SV40-TAg lentivirus. Detection of tumor specific alterations (*BRAF*-duplication and *BRAF V600E*-mutation) by digital droplet PCR (ddPCR) at defined time points allowed for determination of tumor cell fraction (TCF) and evaluation of the workflow. DNA-methylation profiling and gene-panel sequencing were used for molecular profiling of primary samples.

**Results:**

Primary cell suspensions had a mean TCF of 55% (+/− 23% (SD)). No sample in NSM (0/18) and ten samples in ABM (10/18) were successfully transduced. Three of these ten (30%) converted into long-term pLGG cell lines (TCF 100%), while TCF declined to 0% (outgrowth of microenvironmental cells) in 7/10 (70%) cultures. Young patient age was associated with successful model establishment.

**Conclusion:**

A subset of primary PA cultures can be converted into long-term cell lines using SV40-TAg depending on sample intrinsic (patient age) and extrinsic workflow-related (e.g. type of medium, successful transduction) parameters. Careful monitoring of sample-intrinsic and extrinsic factors optimizes the process.

## Introduction

Low-grade gliomas (pLGG) are the most common pediatric brain tumors and account for about 30% of all pediatric brain tumors [1]. pLGG, commonly displaying a hallmark MAPK activation, comprise a set of different WHO grade I and II entities [2]. As in other fields of cancer research, pLGG cell lines suitable for *in-vitro* preclinical experiments are needed for drug development and in-depth mechanistic understanding. In sharp contrast to the high incidence of pLGG, in particular of pilocytic astrocytomas (PA; WHO grade I), the most common subgroup of pLGG, the number of available reliable patient-derived preclinical models is low [2, 3]. Only a handful of patient-derived pLGG *in-vitro* models have been published. The BT-40 cell line [4] harbors a *BRAF V600E*-mutation and a *CDKN2A*-deletion and molecularly reflects a pleomorphic xanthoastrocytoma (PXA) WHO grade II. The PA patient-derived cell line Res186 [5] was shown to have a *PTEN*-deletion but no MAPK-alteration, findings untypical for PA. The recently published cell line JHH_NF1_PA1 [6], derived from a 14-year-old NF1-patient´s PA, was established using a conditional reprogramming cell culture approach to sustain growth of senescence-prone cells. Loss of *NF1*, absence of atypical alterations (such as *ATRX*-loss or *CDKN2A*-loss) and genetic stability over time support JHH_NF1_PA1 to be a true PA model. Other approaches to culture patient-derived pLGG cells beyond classical culture methods include mouse brain-slice overlay cultures [7] or synthetic extracellular matrices with astrocytes [8]. These latter techniques amend the portfolio of preclinical pLGG models but are not ideally suitable for large-scale comprehensive preclinical testing.

A main reason for the failure of PA-cultures in conventional cell culture approaches adapted from higher-grade tumors [6] is oncogene-induced senescence (OIS), described in primary PA and PA short-term cultures [9]. To surmount the obstacle of pLGG paucity we used Simian Virus 40 Large T antigen (SV40-TAg) to reversibly circumvent OIS in primary PA cultures [10]. The generated patient-derived PA cell line, DKFZ-BT66, turned out to be a valuable tool for preclinical drug testing and studies on OIS [11–13]. DKFZ-BT66 was the proof of concept that pLGG-model generation from primary material using SV40-TAg is feasible. However, the success of generating PA cell lines using SV40-TAg was not easily repeated. To improve the model establishment efficiency, we sought to identify factors that influence the success of generating PA cell lines using SV40-TAg, and investigated sample intrinsic (quality of the sample; e.g., sample size, origin, tumor cell content) and extrinsic (quality of the workflow, e.g., culture conditions, enrichment for tumor cells, efficient viral transduction) factors. To this aim, we standardized the modeling workflow and implemented ddPCR, a technique that only requires minimum amounts of genomic DNA input [14], as a tool to closely monitor each step of the culturing process. Here, we report on the performance and outcome of this workflow as well as factors influencing modeling success.

## Materials and methods

### Patient samples

Primary pLGG tumor material was collected during therapeutic surgical intervention in Heidelberg or Tübingen, Germany. Samples were transferred into shiping vials containing unsupplemented NeurobsaI™ medium (21,103,049, Gibco) and stored at 4 °C before processing. Samples from Tübingen were shipped overnight at room temperature. Informed consent for sample collection, use of material and clinical data was obtained within the study S-304/2014 (V2/V3), approved by the institutional review board of the University of Heidelberg. The primary patient tumor material was molecularly analyzed (DNA-methylation and gene panel sequencing) within the PTT2.0 study [15] or the LOGGIC Core co-clinical biobank [16]. Methylation scores were obtained via the brain tumor classifiers (V11b4, 12.3 or 12.5) (www.molecularneuropathology.org).

### Primary culture and cell lines

Processing of primary samples and HEK293T cells was described before [17]. Fully supplemented Astrocyte Basal Medium (ABM) [17] or Neural Stem Cell Medium (NSM) (500 ml DMEM/F12 (1:1) + GlutaMAX (31331-028, Gibco) supplemented with 10ml B27-Supplement w/o vitamine A (12587-010 Gibco), 20ng/ml Human bFGF (100 µg/ml in PBS; AF-100-18B Peprotech) and 20ng/ml Human EGF (100 µg/ml in PBS; AF-100-15 Peprotech) was used for primary culture.

### A2B5 selection

A2B5 positive selection was performed following manufacturer´s instructions with anti-A2B5 MicroBeads (human, mouse, rat; #130-093-392) on a MACS® MultiStand (#130-042-303) using MS Columns (#130-042-201), all from Miltenyi Biotec, Germany. Prior to magnetic separation, tumor cell suspensions were treated with 20 µl of FcR Blocking Reagent (#130-059-901) for 10 min and subsequently with 20 µl of Anti-A2B5 MicroBeads for 15 min. After magnetic separation, cells (from both fractions, positive-selected and flow through, respectively) were centrifuged at 300×g for 10 min, immediately resuspended in culture medium (ABM or NSM) and transferred to a 37.0 °C incubator with humidified atmosphere and 5% CO_2_.

### pCW57.1 dsGFP-TAg plasmid, virus production and lentiviral transduction

The generation of the doxycycline-inducible lentiviral expression vector for expression of SV40 large T (SV40-TAg), pCW57.1 dsGFP-TAg, and generation of viral supernatant was described in detail before [17]. Virus titers were determined after infection of HEK293T cells with serial dilutions of the viral supernatants by counting GFP or RFP positive colonies. Primary pLGG cultures were incubated with pure viral supernatant for six hours before top-up with target cell medium (ABM or NSM). Medium change to full target cell medium was performed after 24 h. The maximum number of sequential infections per sample and condition was three.

### gDNA extraction and ddPCR

Genomic DNA of pLGG tumor cell suspensions was extracted with the QIAamp DNA Mini Kit (Qiagen; #51304). DNA was eluted with 20 µl nuclease-free water.

ddPCR for the detection of the *BRAF*-duplication and the *BRAF V600E*-mutation was performed as described before [14, 17]. Determination of tumor cell fraction (TCF) based on ddPCR results was done as follows 1) *BRAF*-duplicated tumors: BRAF-duplicated tumor cells have a BRAF exon 14 copy number of 3. Microenvironmental non-tumor cells have a BRAF exon 14 copy number of 2. The mean copy number of BRAF exon 14 in a given mixture of tumor cells and non-tumor cells the is, depending on the TCF, between 2 and 3. Accordingly, the TCF could be calculated using the formula:

$$2 - BRAF\,exon\,14\,copy\,number$$

The copy number of BRAF exon 14 was calculated using the ratio of BRAF exon 14 copies (duplicated in case of BRAF-duplication) to BRAF exon 3 (reference; not duplicated in case of BRAF-duplication) using the formula:

$$\frac{{Concentration\,BRAF\,exon\,14\,\left[ {copies/\mu l} \right]}}{{Concentration\,BRAF\,exon\,3\left[ {copies/\mu l} \right]}}\, \cdot 2$$

2) *BRAF V600E*-mutation: The calculation of TCF in a *BRAF V600E*-positive tumor sample assumed heterozygosity of the BRAF-V600E mutation. It was calculated using the ratio of the concentration of *BRAF V600E copies to the concentration of BRAF wildtype (WT) copies.* applying the following formula:

$$\frac{{\frac{{Concentration\,BRAF\,V600E\,\left[ {\frac{{copies}}{{\mu l}}} \right]}}{{Concentration\,BRAF\,WT\,\left[ {\frac{{copies}}{{\mu l}}} \right]}}\, \cdot \,2}}{{\left( {1 + \,\frac{{Concentration\,BRAF\,V600E\,\left[ {\frac{{copies}}{{\mu l}}} \right]}}{{Cconcentration\,BRAF\,WT\,\left[ {\frac{{copies}}{{\mu l}}} \right]}}} \right)}}$$

To calculate the TCF in % the TCF determined using the formulas above was multiplied by 100.

### Graphs, flow-charts and statistics

Flow-diagrams were generated with Biorender (https://www.biorender.com) using the Hopp Children´s Cancer Center´s institutional account. Differences between two groups were compared in R Studio (R Version 4.1.0) using an unpaired *t* test in the “stats” package. Comparisons between multiple groups were done using one-way ANOVA followed by Tukey’s ‘Honest Significant Difference’ test in the “stats” package. Graphs were generated using R package “ggplot2” (v 3.3.5). Boxplots visualize the minimum value, the first quartile (25th percentile), the median, the third quartile (75th percentile) and the maximum value. 

## Results

### Description of sample cohort

Between 02/2020 and 04/2021, 18 primary pLGG samples were included. The clinical and molecular characteristics are summarized in Table 1. The patient age at sampling was 7.9 (+/− 5.4) years (mean+/− SD). PA was the predominant histological diagnosis. Molecular data was available for 17/18 (94%) samples. DNA-methylation analysis confirmed and refined the histopathological diagnoses in 16/18 (89%) cases (either clear molecular diagnosis with scores > 0.9 or highest score for the respective methylation class within the classifier). The most frequent MAPK-alteration found in the cohort were *KIAA1549:BRAF*-fusion (11/18; 61%) and *BRAF V600E*-mutation (3/18; 17%).

**Table 1:** Clinical and molecular features of pLGG samples

ID	Sex	Age at sampling (years)	Histology	Localization	Methylation class (classifier version)	Classifier score	BRAF alteration (method of detection)
pLGG1	F	5	Pilocytic astrocytoma	Posterior fossa	PA_PF (V11b4)	0.99	KIAA1549:BRAF-fusion (DNA-methylation)
pLGG2	F	7	Pilocytic astrocytoma	Posterior fossa	PA_INF (V11b4)	0.99	KIAA1549:BRAF-fusion (DNA-methylation)
pLGG3	F	0	Pilocytic astrocytoma	Optic pathway	PA_MID (V11b4)	0.99	BRAF V600E-mutation (GPS; NPHD 2019 A)
pLGG4	M	5	Pilocytic astrocytoma	Posterior fossa	PA_INF (V12)	0.99	KIAA1549:BRAF-fusion (DNA-methylation)
pLGG5	M	9	Pilocytic astrocytoma	Posterior fossa	PA_INF (V12)	0.94	BRAF V600E-mutation (GPS; NPHD 2019 A)
pLGG6	M	14	Pilocytic astrocytoma	Midbrain	PA_INF (V12)	0.96	KIAA1549:BRAF-fusion (DNA-methylation)
pLGG7	M	2	Pilocytic astrocytoma	Cerebellum	PA_PF (V11b4)	0.99	KIAA1549:BRAF-fusion (DNA-methylation)
pLGG8	F	18	Pilocytic astrocytoma	Posterior fossa	PA_PF (V11b4)	0.99	BRAF 599TT-insertion (GPS; NPHD 2019 A)
pLGG9	M	3	Pilocytic astrocytoma	Posterior fossa	PA_PF (V11b4)	0.99	KIAA1549:BRAF-fusion (DNA-methylation)
pLGG10	M	8	Pilocytic astrocytoma	Parietooccipital	PA_CORT (V12)	0.97	COPC:ROS1-fusion (DNA-methylation)
pLGG11	M	8	Pilocytic astrocytoma	Posterior fossa	PA_PF (V11b4)	0.81	KIAA1549:BRAF-fusion (DNA-methylation)
pLGG12	F	11	Pilocytic astrocytoma	Brain stem	Not classifiable	(tSNE close to PA_MID)	BRAF V600E-mutation (gene panel sequencing)
pLGG13	M	16	Low grade glioma	Left temporal	PA_CORT (V12)	1.0	PRKAR2B:BRAF-fusion (DNA-methylation)
pLGG14	F	8	Pilocytic astrocytoma	Optic pathway	PA_MID (V11b4)	0.69	KIAA1549:BRAF-fusion (DNA-methylation)
pLGG15	M	1	Pilocytic astrocytoma	Brain stem	PA_INF (V12)	0.99	KIAA1549:BRAF-fusion (DNA-methylation)
pLGG16	F	5	Pilocytic astrocytoma	Posterior fossa	PA_PF (V11b4)	0.99	KIAA1549:BRAF-fusion (DNA-methylation)
pLGG17	F	14	Pilocytic astrocytoma	Spinal	Not done	/	BRAF-duplication (ddPCR)
pLGG18	F	14	Pilocytic astrocytoma	Posterior fossa	PA_INF (V12)	0.86	KIAA1549:BRAF-fusion (DNA-methylation)

*F* female, *M* male, *PA*_*PF* posterior fossa pilocytic astrocytoma, *PA*_*INF* infratentorial pilocytic astrocytoma, *PA*_*MID* midline pilocytic astrocytoma, *PA*_*CORT,* cortical pilocytic astrocytoma, *GPS* gene panel sequencing

### Modeling workflow and evaluation of culture processing

**Fig. 1:**
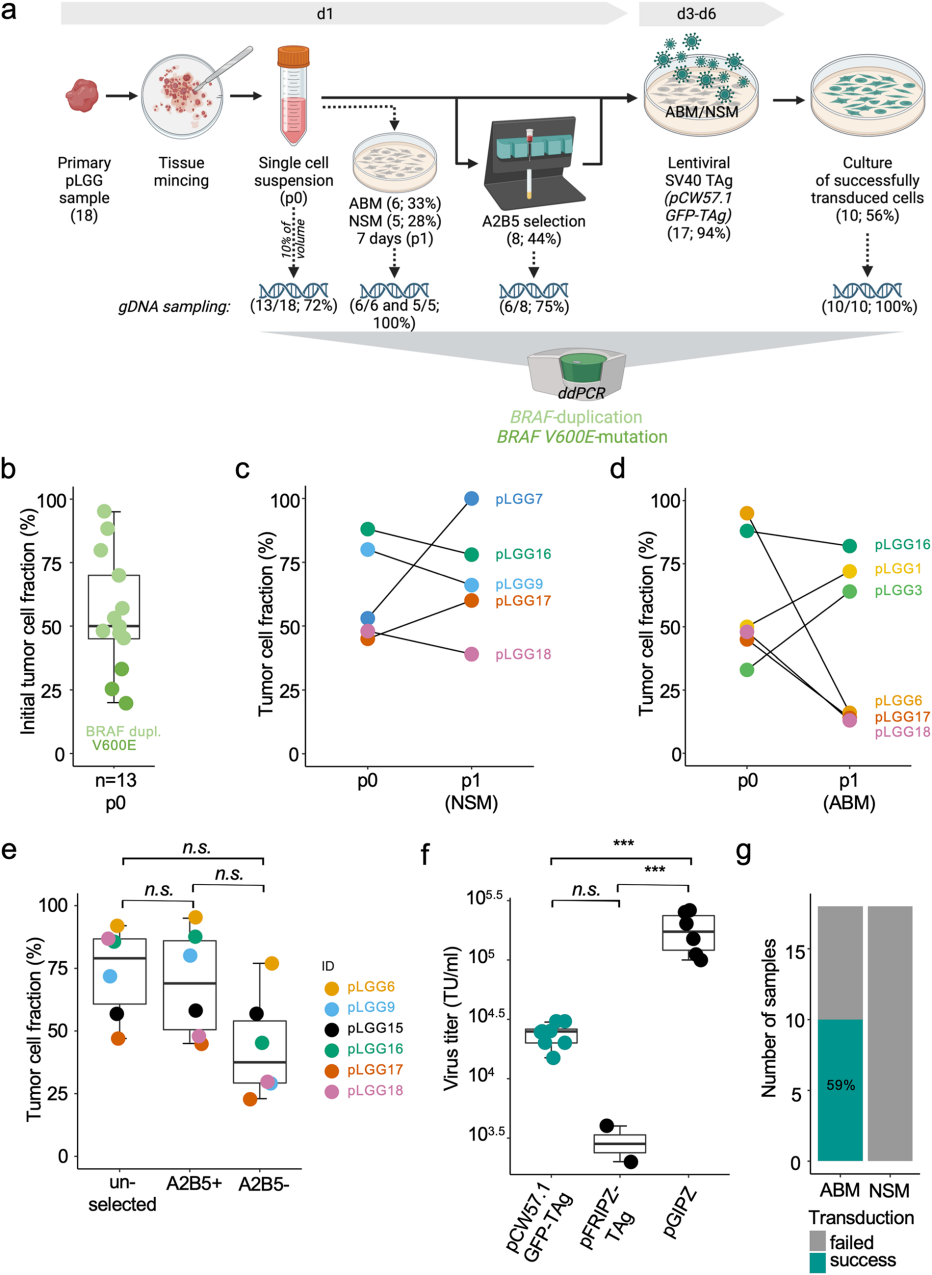
Modeling workflow and evaluation of culture processing **a** Flow-chart depicting the technical modeling process. *gDNA* genomic DNA, *ddPCR* digital droplet PCR, *ABM* astrocyte basal medium, *NSM* Neural stem cell medium. Numbers of samples and percentage (of total or subgroup) in brackets. Created with BioRender.com, **b** Boxplot depicting the initial tumor cell fraction of p0 primary cell suspension of 13 samples, where gDNA was available, **c** comparison of tumor cell fraction in samples of initial primary cell suspension (p0) to corresponding sample after culture for one week in neural stem cell medium (NSM). Data of five tumors **d** Comparison of tumor cell fraction in samples of initial primary cell suspension (p0) to corresponding sample after culture for one week in astrocyte basal medium (ABM). Data of six tumors **e** Boxplot comparing the initial tumor cell fraction (unselected) of six samples to the fraction after A2B5 selection (positive selection fraction; A2B5+) and the flow-through (negative selection fraction; A2B5−) n.s. = not significant (one-way ANOVA followed by Tukey’s ‘Honest Significant Difference’ test). **f** Virus titers obtained 48 h after transfection of HEK293T cells with the plasmids, pCW57.1GFP-TAg, pFRIPZ-TAg and pGIPZ, respectively. *** = adjusted p-value < 0.001, n.s.= not significant (one-way ANOVA followed by Tukey’s ‘Honest Significant Difference’ test). TU: transducing units, **g** Bar graph depicting the fraction of successfully transduced samples and the fraction of samples where transduction failed in the two media, astrocyte basal medium (ABM) and neural stem cell medium (NSM) (**b, e, f** Boxplots visualize the minimum value, the first quartile (25th percentile), the median, the third quartile (75th percentile) and the maximum value)

The workflow of sample processing is shown in Fig. 1a. Single cell suspensions of all tumors were split and cultured in ABM and NSM, respectively. A subset of 8/18 (44%) primary single cell suspensions was submitted to A2B5 selection before seeding. 17/18 (94%) short-term cultures were infected with inducible SV40 TAg after three to six days in culture. gDNA samples were generated, whenever cell numbers allowed, at defined time points and ddPCR was used to detect the two most common MAPK-alterations in pLGG, *BRAF-duplication* and *BRAF V600E* mutation. This allowed for qualitative (tumor cell identity) and quantitative (TCF vs. microenvironmental cell populations) control of the cultures. Of note, three of the primary samples harbored different MAPK-alterations and could therefore not be followed by ddPCR. gDNA samples were taken immediately after dissociation (p0) for 13/18 pLGG tumors (72%) (Fig. 1a), and tumor specific MAPK alterations were confirmed in all 13 samples. The TCF of the initial single cell suspensions was 55 +/− 23% (mean, +/− SD; range: 20–95%) (Fig. 1b).

To investigate the sole effect of culture media on pLGG tumor cell survival, a fraction of primary cells was seeded in an extra dish whenever the amount of primary suspension allowed for. TCF was then assessed by ddPCR after one week of culture (p1; ABM: 6/18; 33%, NSM: 5/18; 28%) (Fig. 1a). Tumor cells could be detected in all tested cases after one week, however there was a sample-dependent change in TCF over time. In some cases, TCF increased over time (e.g. pLGG7 in NSM or pLGG1 and pLGG3 in ABM) while in other cases TCF declined from p0 to p1 (e.g. pLGG6, pLGG17 and pLGG18 in ABM) (Fig. 1c, d).

As the cell surface ganglioside epitope A2B5 is expressed on PA tumor cells [18, 19], we sought to evaluate A2B5 positive selection as a tool to a priori enrich cultures for tumor cells. Comparison of TCF before and after selection (6/18 cases; 33%) could not confirm a significant increase in TCF after positive A2B5 selection (Fig. 1e). However, because a positive control was not included in these experiments, we could not exclude an artefact of technical failure.

Transduction of primary pLGG cells with inducible SV40-TAg was the central modeling tool to enable circumvention of OIS and proliferation. 17 of 18 (94%) short-term cultures were infected with SV40-TAg. In one case (6%) no viable cells were present after seeding. The plasmid pCW57.1 dsGFP-TAg [17] was designed to have a lower size between the long terminal repeats (LTRs) (8.2kbp) compared to the formerly used plasmid pFRIPZ-TAg (11.0 kbp) [10] aiming at a higher lentiviral packaging efficiency. pCW57.1 dsGFP-TAg produced higher viral titers, however they were not significantly different from pFRIPZ-TAg (Fig. 1f) and still significantly lower compared to the control plasmid pGIPZ (6.6 kbp between LTRs). Ten of 17 primary cultures (59%) in ABM could successfully be transduced with pCW57.1 dsGFP-TAg supernatant. However, none of the pLGG cultures in NSM could be successfully transduced (Fig. 1g).

### Modeling outcome

**Fig. 2:**
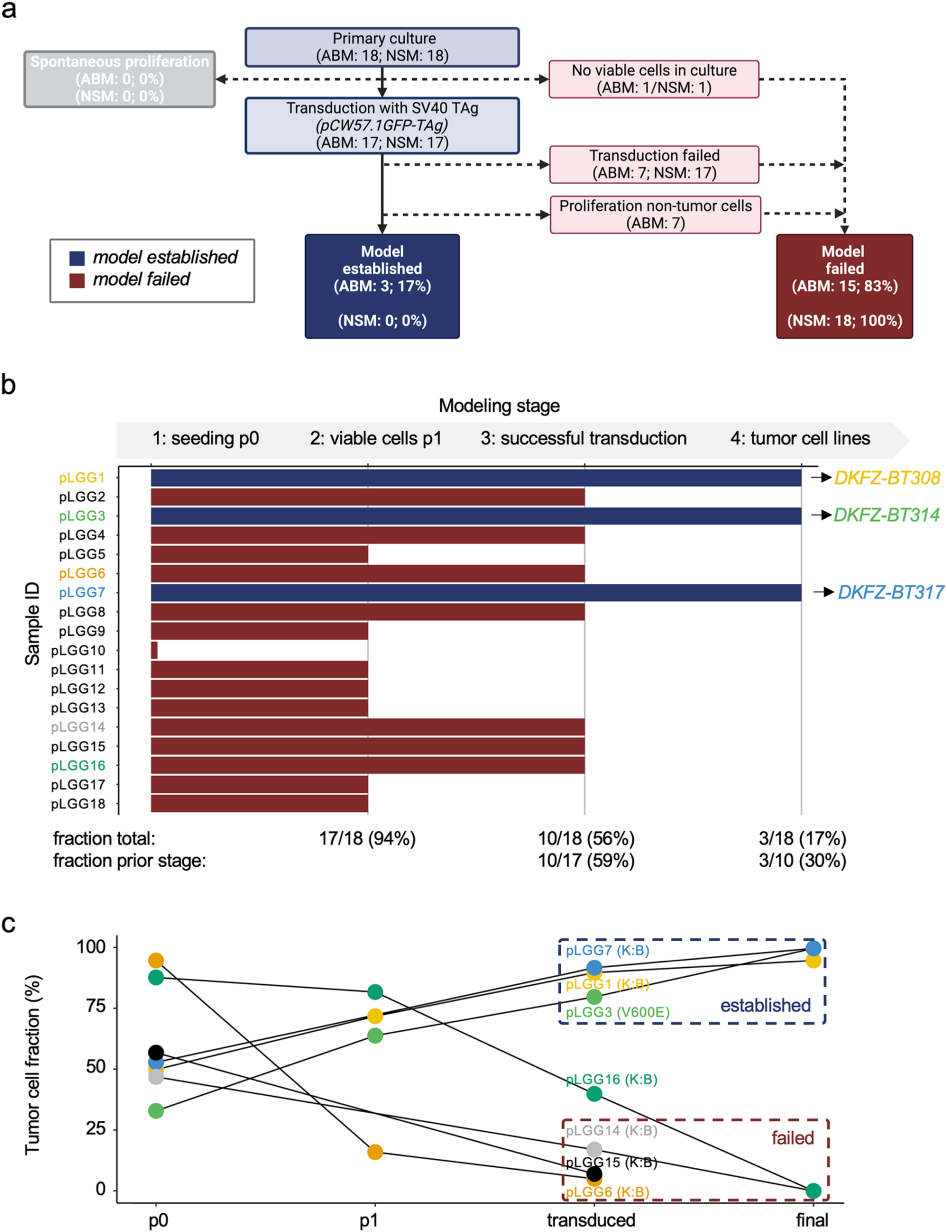
Modeling outcome **a** Flow-chart depicting the overall outcome of the PA -modeling workflow. *ABM* Astrocyte Basal Medium, *NSM* Neuronal Stem-cell Medium. Created with BioRender.com, **b** Swimmer plot depicting the advancement of every single sample in the modeling process, **c** Tumor cell content calculated from ddPCR data of seven samples successfully transduced with SV40 TAg at different time points during the culturing procedure. p0: initial sample; p1: after one week in culture; transduced: first passage after successful infection; final: latest ddPCR performed for the respective sample; (K:B): *KIAA1549:BRAF*-fusion harboring sample; (V600E): *BRAF* *V600E*-mutation harboring sample

The outcome of our modeling workflow is summarized in the flow-chart depicted in Fig. 2a. None of the 18 primary samples gave rise to a spontaneously proliferating tumor cell line without SV40-TAg transduction, in line with prior experiences [6]. None of the samples cultured in NSM were successfully transduced, and thus no long-term cell line was derived from primary cultures in NSM. A total of 3 samples (17%) cultured in ABM could be converted into long-term proliferating PA cell lines following transduction with SV40-TAg. The performance of each individual sample (cultured in ABM) within the process with the levels “viable cells at p1”, “successful transduction” and establishment of “tumor cells lines” is depicted in a swimmer plot (Fig. 2b). Ten of 17 samples (59%) with viable cells at p1 could be successfully transduced with SV40-TAg and 3 of 10 (30%) successfully transduced primary cultures converted into PA tumor cell lines. These lines are fully described and characterized in Selt et al. [17]. and preserve PA-typical molecular and functional characteristics including methylome, genomic stability, expression of tumor-specific MAPK-alterations with MAPK-activation, re-induction of OIS in the absence of SV40TAg followed by senescence features like SA-b-galactosidase positivity and upregulation of OIS/senescence gene sets. For 7 of 10 (78%) successfully transduced samples, longitudinal TCF data across the full modeling process, from p0 (initial sample after dissociation), p1 (after about 1 week in culture), shortly after transduction (“transduced”) to a late time point after transduction (“final”), was available (Fig. 2c). Strikingly, two clear patterns were observed: TCF either increased during the procedure and reached 100% (long-term tumor cell lines established; successful) or decreased and finally reached 0% (no cell lines established; outgrowth of microenvironmental cells; failed).

### Features of established models and possible determinates of modeling success

**Fig. 3:**
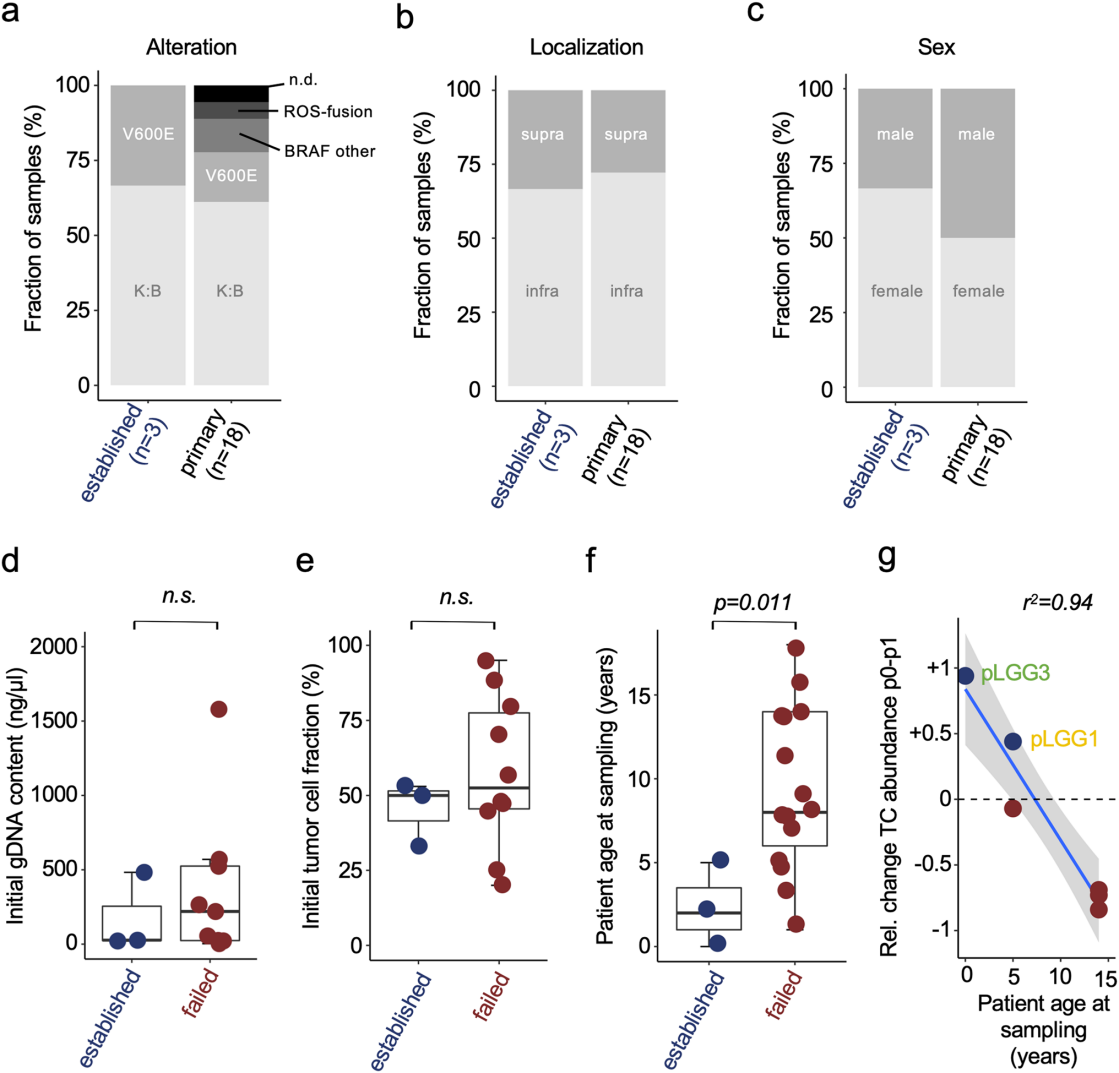
Features of models and possible determinates of modeling success **a**–**c** Distribution of the clinical features genetic alteration (“alteration”) (**a**), tumor localization (“localization”) (**b**) and sex of the patients (“sex”) (**c**) in the cohort of samples that could be successfully transformed into long-term PA cell lines (n = 3; “established”) compared to the complete primary cohort (n = 18; “primary”). K:B: *KIAA1549:BRAF*-fusion; V600E: *BRAF* *V600E*-mutation; BRAF other: BRAF-fusion other than KIAA1549:BRAF-fusion; supra: supratentorial tumor; infra: infratentorial tumor, *n*.*d*. not determined, **d** Boxplot depicting the gDNA concentration of p0 samples as surrogate parameter for initial sample size. Samples that could be transformed into long-term pLGG models (“established”; n = 3) and samples that failed (“failed”; n = 10) were compared, n.s.: not significant, unpaired *t* test p > 0,05 **e** Boxplot depicting the initial tumor cell fraction (in samples used for transduction) in the two groups of samples that either turned into long-term pLGG models (“established”; n = 3) or failed (“failed”; n = 10). *n*.*s*. not significant, unpaired *t* test p > 0.05, **f** Boxplot depicting the patient age at sampling in the two groups of samples that either turned into long-term pLGG models (“established”; n = 3) or failed (“failed”; n = 15); t-test **g** xy-plot showing the parameters patient age at sampling (x) and the relative change in tumor cell fraction from p0 to p1 in ABM medium (y) and the correlation of the two parameters. (**d**–**f** Boxplots visualize the minimum value, the first quartile (25th percentile), the median, the third quartile (75th percentile) and the maximum value)

Successful modeling was not restricted to one of the most common MAPK alterations of our cohort (BRAF V600E-mutation or *KIAA1549:BRAF*-fusions), location of the tumor or sex of the patient (Fig. 3a–c). The gDNA concentration in the standardized sample at p0 (influenced by e.g. tumor sample size, cellular content, tissue quality) (Fig. 3d) and the TCF at p0 (Fig. 3e) were not significantly different between the groups of primary samples that later turned into successful models or failed. However, the patient age at sampling was significantly lower (mean 2.3 years) in the group of successful models compared to the group of samples that failed to convert into cell lines (mean 8.9 years) (Fig. 3f). In a subset of the cohort (6 of 18 samples (33%)) we had enough material to additionally test the ability of ABM to maintain TCF in short-term culture before transduction. Here, patient age was negatively correlated with the relative change in TCF from p0 to p1 in ABM (Fig. 3g), indicating that tumor cells in samples from younger patients had a higher proliferative capacity compared to their microenvironmental cells.

### Recommendations for SV40 TAg-based pLGG cell line establishment

Based on the factors analyzed above we recommend to consider several intrinsic (tumor) and extrinsic (technical) factors to optimize the success in establishing SV40-TAg-based PA cell lines from primary patient material (Table 2). The sample intrinsic factor contributing to a higher likelihood of successful model establishment is patient age. The extrinsic factors contributing to a higher likelihood of successful model establishment are: cell culture medium (e.g., ABM), successful transduction (efficient viral packaging, viral titers), and maintenance of the TCF (monitoring by e.g., ddPCR).

**Table 2:** Factors influencing SV40-TAg based PA cell line modeling success

Factor	Influence on modeling	Considerations
Sample intrinsic factors		
Sex	No	Samples from patients ≤ 5 years of age are more likely to convert into PA tumor cell lines after SV40-TAg transduction. Other factors likely have no major influence
Localization	No	
Age at sampling	Yes	
BRAF-alteration	No	
Sample size	No	
Primary TCF	No	
Extrinsic factors		
Cell culture medium	Yes	Medium must maintain TCF and allow for transduction; here ABM
A priori enrichment for TCF	No	e.g.A2B5 selection - not advisable, because primary TCF is not a prognostic factor
Successful SV40-TAg transduction	Yes	Optimized protocols needed for successful transduction (viral titers, protocols for stem cell conditions, etc.)
Maintenance of the TCF (after transduction)	Yes	Careful monitoring e.g.by ddPCR needed

## Discussion

Expression of SV40-TAg in primary PA cells allows to circumvent OIS and generate proliferating PA cell lines. The resulting PA *in-vitro* models are of high translational significance: As shown before [10, 17], the generated cell lines clearly reflect PA biology, even though they are genetically modified. While expression of SV40-TAg may result in different outcomes (e.g. cell death, apoptosis, transformation or no effect) in different tissues [20], for pLGGs we have previously shown that SV40-TAg overcomes OIS but does not transform or immortalize PA cells. SV40-TAg transduced PA cells have a stable genome, re-enter OIS upon SV40-TAg withdrawal and enter replicative senescence at the end of their lifespan. Unlike any other PA model to date, inducible SV40-TAg models allow to also study the senescent state of the cells after re-induction of OIS. Long-term expandability overcomes the limitation of low proliferation rates and short lifespans observed in other primary modeling approaches [21]. SV40-TAg PA models are less cost effective compared to e.g. cultures in synthetic extracellular matrices [8]. Taken together, they are excellent and unique tools for standardized studies of tumor cell intrinsic mechanisms and preclinical drug testing to uncover new therapeutic approaches, such as rational combination treatments [12] or senolytic BH3-mimetics [17].

Only a fraction of primary PA samples were successfully converted into proliferating tumor cell lines using the SV40-TAg approach in our hands. In the present cohort, monitoring by ddPCR was applied, aiming at a more profound understanding of modeling processes and identification of determinants of success and failure. Of note, the ddPCR assays used detected *BRAF*-duplications and the *BRAF V600E*-mutation. Two of the 18 primary samples (11%) (pLGG8 and pLGG10) had different MAPK-alterations and therefore could not be monitored by ddPCR. Furthermore, due to small sample sizes and resulting low amounts of material, gDNA-samples for ddPCR could not be collected at all intended timepoints for all samples.

ddPCR identified the underlying MAPK-alteration in all 13 tested primary PA suspensions (p0). TCF in the primary suspensions was median 55% and in line with published tumor/microenvironment ratios in PA [18, 22]. This confirmed a high quality of the primary surgical samples and excluded quality of the primary samples as a major factor of modeling success and failure.

As expected, and in line with previous observations [6] none of the 18 primary samples gave rise to a proliferating cell lines without SV40-TAg transduction. Successful transduction was therefore a prerequisite for model generation. The plasmid pCW57.1 dsGFP-TAg yielded titers that were consistent throughout different preparations and therefore unlikely to be a major variable of modeling success. The comparably low titers obtained were most likely due to the large SV40-TAg insert (> 8kbp) which reduces the lentiviral packaging efficiency [23]. The supernatant allowed for successful transduction of 59% of the infected primary cultures in ABM. This rate is lower compared to published studies in glioblastoma for example, where infection was shown to be successful in 100% of primary samples [24]. This difference might be explained by the different biology of pLGG and the aforementioned low viral titers. Only primary cultures in ABM but not under stem cell conditions (NSM) were successfully transduced. Transduction protocols especially adapted to stem cell conditions [25] might therefore be an option to successfully transduce primary pLGG cells cultured in NSM. NSM was not shown to be inferior in its ability to maintain tumor cell survival in short-term culture and successful transduction of these cultures might increase the number of successful models.

Sample intrinsic factors like sex of the patient, tumor localization, genetic alteration or initial size of the sample likely did not influence the modeling success. Initial TCF of the sample was not a factor indicative of later success. A priori enrichment of TCF by e.g. A2B5-selection is therefore not necessary or advisable and we did not focus on improvement of this selection process. The age of the patients at sampling was associated with modeling outcome. Only samples from younger patients (≤ 5 years) generated pLGG-models after infection. In line with this observation, our first reported PA cell line (DKFZ-BT66) was also derived from a young patient (two years) [10]. Of note, a young age alone was not a guarantee for modeling success because some samples from patients of young age also failed. An earlier study had shown that pLGGs from young patients (< 4 years) had significantly longer telomeres compared to older patients (> 10 years) [26] and older patients had lower chance to relapse. Our own previous cell line study proved dependence on replicative senescence in later passages of DKFZ-BT66 despite SV40-TAg expression [10], indicating that SV40-TAg can override OIS but not replicative senescence in PA. This might explain, why model generation based on SV40-TAg expression is more likely to be successful in samples from younger aged patients, because their tumor cells still inherit a higher replicative capacity due to longer telomeres. Moreover, our limited available data indicated a generally stronger ability of tumor cells from younger patients to survive/proliferate short-term in culture without SV40-TAg. This points at a comparably higher intrinsic proliferative capacity in the very first days of culture in younger PA cells. Although lentiviral gene-transfer is known to be successful in non-dividing cells [27] the higher proliferative capacity of younger PA cells in turn might create a more susceptible state for successful transduction and outgrowth of PA tumor cells over co-transduced microenvironmental cells in the primary culture.

Taken together, based on the data presented in this study patient age is the only major sample intrinsic factor influencing success rate of PA cell line generation using SV40-Tag. Several extrinsic factors contributing to a higher likelihood of successful model establishment, like type of medium, efficacy of transduction and maintenance of TCF, should be considered to optimize the outcome. Monitoring of PA cultures by ddPCR is strongly recommended to control for quality.
